# Memory Storage Fidelity in the Hippocampal Circuit: The Role of Subregions and Input Statistics

**DOI:** 10.1371/journal.pcbi.1004250

**Published:** 2015-05-08

**Authors:** Torsten Neher, Sen Cheng, Laurenz Wiskott

**Affiliations:** 1 International Graduate School Neuroscience, Ruhr-University Bochum, Bochum, Germany; 2 Institute for Neural Computation, Ruhr-University Bochum, Bochum, Germany; 3 Mercator Research Group ‘Structure of Memory’, Department of Psychology, Ruhr-University Bochum, Bochum, Germany; SISSA Intl Sch Adv Studies, ITALY

## Abstract

In the last decades a standard model regarding the function of the hippocampus in memory formation has been established and tested computationally. It has been argued that the CA3 region works as an auto-associative memory and that its recurrent fibers are the actual storing place of the memories. Furthermore, to work properly CA3 requires memory patterns that are mutually uncorrelated. It has been suggested that the dentate gyrus orthogonalizes the patterns before storage, a process known as pattern separation. In this study we review the model when random input patterns are presented for storage and investigate whether it is capable of storing patterns of more realistic entorhinal grid cell input. Surprisingly, we find that an auto-associative CA3 net is redundant for random inputs up to moderate noise levels and is only beneficial at high noise levels. When grid cell input is presented, auto-association is even harmful for memory performance at all levels. Furthermore, we find that Hebbian learning in the dentate gyrus does not support its function as a pattern separator. These findings challenge the standard framework and support an alternative view where the simpler EC-CA1-EC network is sufficient for memory storage.

## Author Summary

It is well known that the hippocampus, a mammalian brain region, has a crucial role in memory formation. Furthermore, it has a remarkable anatomical structure and can be divided into several subregions based on physiological properties. Over the last decades a widely accepted model has evolved suggesting individual roles for each subregion in memory storage. The central idea is that region CA3, with its remarkably many synapses that project into the region itself again, stores the memories within these synapses. In this model a memory is impressed onto the hippocampus by neuronal activation in the hippocampal input region. Recently it has been found that such activations have a certain regularity instead of being random as assumed in the standard model. Here we investigate how well the model performs when storing memories of regular inputs. We find that the proposed function of CA3 actually harms memory performance. Moreover, we show that this function is redundant even in the case of random inputs. These findings call the standard model into question and support an alternative view of how the hippocampus may store memories.

## Introduction

The crucial role of the hippocampus in memory formation is well known. Patients with damage to the hippocampus and its nearby cortices have severe deficits in acquiring new episodic memory and in remembering events that happened shortly before the damage [1]. Impairments in memory formation can be observed in animals, too. For example, rats with a lesioned hippocampus cannot associate stimuli if there is a time delay between them [2].

Furthermore, the hippocampus has a remarkable anatomical structure. Based on anatomical and physiological properties it can be divided into the dentate gyrus (DG) with its huge number of small granule cells that show low activity [3] and the regions CA3 and CA1 consisting of a homogeneous set of pyramidal cells, where in CA3 one can find a striking number of recurrent connections. A further notable property is that the connections among the subregions is established largely in a feedforward manner [4].

The question that arises is, how does this peculiar anatomical structure serve memory formation? Over the years, a standard framework has evolved regarding hippocampal functioning and it has been tested with a number of computational models (for example by Rolls (1995) [5]). The view is that the CA3 region functions as an auto-associative memory [6–9]. An auto-associative memory is a recurrent network that stores patterns in its feedback connections and can reconstruct these patterns when only a partial version of them is presented. Thus, the actual storing place are the recurrent connections and this idea could explain why there are so remarkably many in CA3.

An auto-associative memory can only store patterns that are not similar or mutually correlated [6]. By nature, however, the neural activation in the input region of the hippocampus, the entorhinal cortex (EC), is not uncorrelated [10]. Thus, it has been suggested that the DG performs pattern separation during the storage phase [7–9]. It decorrelates the patterns of the EC and projects the separated versions of the patterns to CA3 for storage. A large number of cells with low activity and the sparse projection of mossy fibers support pattern separation computationally [11, 12]. Hence, this view explains the appearance of yet other prominent hippocampal characteristics. Finally, it has been proposed that the role of CA1 is to decode the highly transformed patterns in CA3 back to their original versions in the EC.

A number of cells in the medial entorhinal cortex (MEC), called grid cells, fire at places in the environment regularly distributed on a hexagonal grid [10]. The present study reviews the model by Rolls and we test whether it is capable of storing grid cell input patterns. To our surprise, we find that CA3 functioning as an auto-associative memory is not only redundant, but harmful for memory performance. We even find a redundancy for random inputs at low to moderate noise levels. Moreover, pattern separation through the DG does not occur by simply applying Hebbian learning.

Since it was recently suggested that the subnetwork EC-CA1-EC already performs pattern completion [13], we tested whether this simpler network is indeed sufficient for memory function.

## Materials and Methods

### Model Architecture and Activation Function

**Fig 1:**
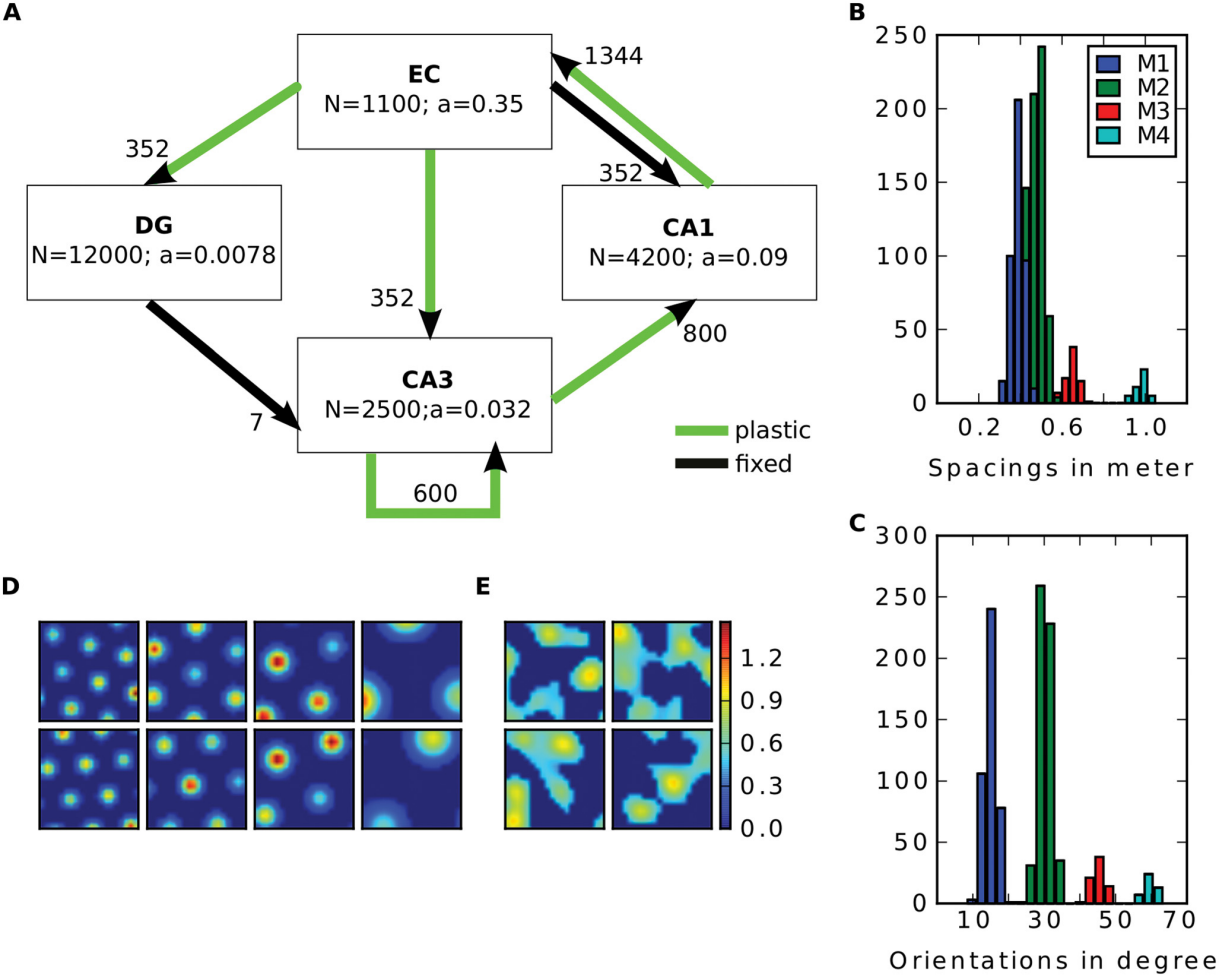
Model overview. **A**: The four subregions EC, DG, CA3 and CA1 are modeled. *a* denotes the proportion of cells being active at any given time. Arrows indicate connectivity among regions. Black ones are random and fixed connections, green ones are plastic and adjusted during learning. The number next to the arrows show the number of connections one cell in the downstream region has with the up stream region. **B-C**: Distribution of spacings (**B**) and orientations (**C**) of the grid population in one environment. Colours indicate the modules. **D-E**: Four examples of grid cells (one from each module) (**D**) and two examples of LEC cells (**E**). The two rows show the firing map of the cells in two distinct environments.

The model consists of the subregions entorhinal cortex (EC), dentate gyrus (DG), CA3 and CA1. Cell numbers *N* in each region and numbers of connections one cell in a downstream region has with the upper region are summarized in Fig 1. Cell numbers and numbers of connections are derived from rat data [4, 14] and scaled down by 100 and 10, respectively. Dividing the number of connections per cell by 100, too, would lead to CA3 cells that do not receive any input from the DG. On the other hand, leaving this number constant would result in triple connections among cell pairs in the network. Thus, we choose to scale by the square root of 100, which scales the total number of connections between two subregions by 100, too. Cells in our model have continuous firing rates with the exception of CA3 cells, which are binary, i.e., they either fire and have the value 1 or are silent and have the value 0. This is in line with Rolls (1995), where CA3 does not work well with continuous firing rates [5].

A pattern **p** of neural activation, for example, $\text{p}\in {\mathbb{R}}_{+}^{N_{EC}}$ in the EC triggers neural activity in a downstream region, e.g., in the DG, via the connections as follows: First, the activation *h*_*i*_ of the output cell *i* is calculated by the standard weighted sum of its inputs

$$\begin{array}{c} h_{i}=\sum_{j=1}^{N_{EC}}w_{ij}p_{j}, \end{array}$$ (1)

where *w*_*ij*_ is the strength of the connection from cell *j* to cell *i* and is defined as 0 whenever this connection is not existent.

To determine the firing of a cell a simple *k*-Winner-Take-All (WTA) mechanism is applied: After calculating the activation of all cells of that region, the *k* cells with the highest activation are either set to 1 or to *h*_*i*_ whenever they are continuous. The others are inhibited and set to 0. The number *k* is determined by the sparsity *a* of that region, i.e *k* = *aN*. For instance, the pattern of neural activity $\text{q}\in {\mathbb{R}}_{+}^{N_{DG}}$ in the DG is

$$\begin{array}{c} q_{i}=\{\begin{array}{cc} h_{i} & \;\text{if}\;h_{i}\;\text{is}\;\text{among}\;\text{the}\;\text{k}\;\text{highest}\;\{h_{j}:1\leq j\leq N_{DG}\}\\ 0 & \;\text{otherwise}. \end{array} \end{array}$$ (2)

Thus, inhibitory cells are not modeled explicitly but rather through their effect on a population level [15–19].

In order to determine the sparsity *a* we first estimated the average number of cells being active in one environment by referring to several studies that count active cells by immediate early genes [20–23] or by electrophysiological recordings [24, 25]. Individual reports are summarized in Table 1 and yield average activity levels of 2.9% in the DG, 22.7% in CA3 and 42.7% in CA1 across the enclosure. Second, for simplicity we assume that every active cell is a place cell and the proportion of the environment a place cell fires in is determined by its place field size times the number of fields the cell has. Using data from recordings within a 1m^2^ apparatus [24, Supplementary Table 1], we obtain an average coverage of 14% of a CA3 cell, and 21% of a CA1 cell. A typical DG cell has 3–4 fields and a field size smaller than 900cm^2^ (personal communication with Edvard Moser) which brings us to an estimation of 27% coverage. Multiplying the proportion of cells being active across the environment by the proportion of the environment one active cells fires leads to the activation level at one location given by *a* (see Fig 1). For the EC we calculated the average coverage of a grid cell to be 35% using data from Hafting et al. (2005) and assume that a grid cell is active in every environment [10, 26]. This value is similar to the value obtained by the simulations from others [27].

**Table 1:** Overview of measured activity levels in hippocampal subregions.

Study	Method	Active cells %
**DG**		
[23] (Fig 3)	IEG	3
[22] (Fig 5)	IEG	3–4
[21] (Fig 7)	IEG	2.2
**CA3**		
[20] (Fig 3c)	IEG (Arc, Homer1)	18
[24]	Electrophysiology	17–32
[25]	Electrophysiology	26
**CA1**		
[20] (Fig 3c)	IEG (Arc, Homer1)	35
[24]	Electrophysiology	48–66
[25]	Electrophysiology	36

IEG means immediate early genes

### Learning Rules

To store patterns in the network the plastic weights among subregions (green arrows in Fig 1) are adjusted by three related Hebbian learning rules. Let **C** denote the connection matrix of two regions, i.e., *c*_*ij*_ = 1 if there is a connection from cell *j* to *i* and *c*_*ij*_ = 0 otherwise.

For the connections EC to CA3, CA3 to CA1, and CA1 to EC a rule for hetero-association is used. Let {**p** ^(*s*)^:1 ≤ *s* ≤ *M*} be the set of *M* input patterns and {**q** ^(*s*)^:1 ≤ *s* ≤ *M*} be the set of output patterns, then the connection strength is defined according to the so called Stent-Stinger rule [28]

$$\begin{array}{c} w_{ij}=c_{ij}\sum_{s=1}^{M}\left(p_{j}^{\left(s\right)}-{\bar{p}}_{j}\right)q_{i}^{\left(s\right)}, \end{array}$$ (3)

where the connection from cell *j* to *i* is the sum over all patterns *s* of firing $p_{j}^{\left(s\right)}$ of input cell *j* subtracted by its mean ${\bar{p}}_{j}$ times the firing $q_{i}^{\left(s\right)}$ of cell *i*. The factor *c*_*ij*_ assures that non-existing connections remain at zero weight.

For the synaptic weight matrix **V** of the recurrent weights in CA3 the co-variance rule is used [29] to learn an auto-association among a set of patterns {**p** ^(*s*)^:1 ≤ *s* ≤ *M*}

$$\begin{array}{c} v_{ij}=c_{ij}\sum_{s=1}^{M}\left(p_{j}^{\left(s\right)}-{\bar{p}}_{j}\right)\left(p_{i}^{\left(s\right)}-{\bar{p}}_{i}\right). \end{array}$$

By subtracting the mean the two learning rules model LTP and LTD. Furthermore the subtraction is essential for computational reasons (see for example [30, chapter 8.2]).

Finally, the connections from EC to DG are altered by a one shot competitive learning rule. Here, the current input pattern **p** first triggers a firing pattern **q** in the downstream region according to the equations above. Synapses are then changed by

$$\begin{array}{c} w_{ij}=c_{ij}\left(w_{ij}^{old}+\gamma p_{j}q_{i}\right), \end{array}$$ (4)

where *γ* is a constant learning rate. After applying Eq (4) the Euclidean norm of vector **w**_*i*_ of incoming weights to cell *i* is normalized to one to assure that not always the same cells get activated. These rules are adopted from Rolls (1995) to keep the model as similar as possible to that one.

After hetero-association of {**p** ^(*s*)^:1 ≤ *s* ≤ *M*} with {**q** ^(*s*)^:1 ≤ *s* ≤ *M*} by applying Eq (3) between some regions, given pattern **p** ^(*t*)^ as the present input we can rewrite the activation $h_{i}^{\left(t\right)}$ as

$$\begin{array}{ccc} h_{i}^{\left(t\right)}\; & = & \;\sum_{j=1}^{N}w_{ij}\;p_{j}^{\left(t\right)}\\ & \;\overset{\left(3\right)}{=}\; & \sum_{j=1}^{N}c_{ij}\sum_{s=1}^{M}\left(p_{j}^{\left(s\right)}-{\bar{p}}_{j}\right)q_{i}^{\left(s\right)}\;p_{j}^{\left(t\right)}\\ \; & = & \;q_{i}^{\left(t\right)}\sum_{j=1}^{N}c_{ij}\left(p_{j}^{\left(t\right)}-{\bar{p}}_{j}\right)\;p_{j}^{\left(t\right)}+\sum_{s\neq t}q_{i}^{\left(s\right)}\sum_{j=1}^{N}c_{ij}\left(p_{j}^{\left(s\right)}-{\bar{p}}_{j}\right)\;p_{j}^{\left(t\right)}\\ & \;\approx & \;q_{i}^{\left(t\right)}\;\underset{S_{t}}{\underset{︸}{c\;{\left(p^{\left(t\right)}-\bar{p}\right)}^{T}p^{\left(t\right)}}}+\sum_{s\neq t}\underset{X_{\left(i,s,t\right)}}{\underset{︸}{c\;q_{i}^{\left(s\right)}{\left(p^{\left(s\right)}-\bar{p}\right)}^{T}p^{\left(t\right)}}}, \end{array}$$

where *c* is the proportion of cells one output cell is connected to in the input layer. Thus, we can write the activation of cell *i* as the sum of a signal term *q*_*i*_ *S*_*t*_ which stems from the proportion of the weights arising from the storage of pattern **p** ^(*t*)^ and the crosstalk terms *X*_(*i*,*s*,*t*)_ which come from the contribution of the other stored patterns in which this cell was active [31]

$$\begin{array}{ccc} h_{i}^{\left(t\right)} & \approx & q_{i}^{\left(t\right)}S_{t}+\sum_{s\neq t}X_{\left(i,s,t\right)}. \end{array}$$ (5)

Ideally, the activation is high if and only if the cell has fired in pattern **q** ^(*t*)^.

### Input

We perform simulations with random patterns and more realistic patterns that were generated by a grid cell code in the medial entorhinal cortex (MEC). For a randomly created pattern, cell activity *h*_*i*_ is sampled from a normal distribution with mean and variance equal to 1. All cells, but the *k* ones with the highest activation are set to zero, as in Eq (2). We store 252 patterns in both cases.

For the grid cell input we built a 1m by 1m virtual square environment. Each cell is equipped with a hexagonal grid of place fields with equal size. The peak firing rate of each field is drawn from a uniform distribution from 0.5 to 1.5. According to the findings of Stensola et al. (2012) we divided the grid cell population into four modules [32]. Cells belonging to the same module have similar grid spacing and orientation. The cell parameter are drawn from normal distributions with variances 8 cm and 3 degree, respectively. The mean spacings of the modules are 38.8, 48.4, 65 and 98.4 cm and are taken from [32, Fig 1D]. The mean orientations are 15, 30, 45 and 60 degrees. The resulting distribution of spacings and orientations of the population is illustrated in Fig 1B–1C. As in Stensola et al. (2012) there are more cells in the modules with small spacings.

Activation of cell *i* at one location is determined by

$$\begin{array}{c} h_{i}=e^{-{\left(\frac{d}{r_{i}}\right)}^{2}log\left(5\right)}\cdot p_{j}^{\left(i\right)}, \end{array}$$

where *d* is the Euclidean distance to the nearest field center *j* and $p_{j}^{\left(i\right)}$ is the peak rate in that field and *r*_*i*_ is the radius. This way its activation is $p_{j}^{\left(i\right)}$ at the center and $0.2p_{j}^{\left(i\right)}$ at the border of one field, which is motivated by the definition of a place field [10]. We measure the activity of all cells at 400 locations uniformly distributed throughout the space. At each location all cells but the *k* ones with the highest activation are set to zero as in Eq (2). For storage, 252 out of the 400 locations are chosen randomly and the corresponding population activities were considered as input patterns.

To study the influence of the lateral entorhinal cortex (LEC) we add input from LEC in some simulations. The activation map of one LEC cell is the summation of 30 place fields with random size at random locations. This is inspired by the findings of Deshmukh and Knierim (2011). They show that cells in the LEC tend to have several pseudo place fields that actually code for specific objects [33]. In Rennó-Costa (2010) LEC cells are modelled similarly. There, the cell’s activation map has specific active and non-active regions [15]. After defining the map in our model, we again set all LEC cells but the *k* ones with the highest activation in each location to zero as in Eq (2).

To study the effect of global remapping, input patterns from different environments are stored in some simulations. Here, each input cell has an activation map for each environment. For a grid cell, its activation map is computed by rotating and shifting its grid structure defined in the first environment, where the rotation angle and shifting vector is the same for the cells from the same module. This is inspired by the results of Fyhn et al. (2007), where they find a coherent remapping in cells recorded at the same location in the MEC [26]. For an LEC cell we define a completely new map for each environment in the same way as for the first map. Examples of input cells and their remapping are shown in Fig 1D–1E.

For recall a noisy version of a stored pattern is created, which we call recall cue. For each noisy pattern a subset of cells is selected randomly to fire incorrectly by setting its rate to that of an arbitrary other cell in that pattern. The quality of the cue is controlled by the number of cells that fire incorrectly and is measured by the Pearson correlation between original pattern and the recall cue.

### Storage and Recall

Storing a pattern **p** of entorhinal activation in the network is done as follows. First, this pattern triggers neural activity in the DG which in turn triggers a pattern in the CA3 region via Eqs (1) and (2). Thus, during storage, activity in CA3 is only influenced by the mossy fiber input from the DG. The connections from EC to DG are altered by the competitive learning rule (Eq (4)) for pattern separation. Hence, for the next pattern the connections are different as for the current pattern. Furthermore, **p** drives an activity pattern in CA1. Now, the pattern in CA3 is hetero-associated with **p** in EC, auto-associated in the recurrent connections in CA3, and hetero-associated with the pattern in CA1. Finally, the CA1 activity is hetero-associated with **p** in the EC.

After the storage of all patterns the network is presented a recall cue by setting entorhinal activity to a noisy version $\hat{\text{p}}$ of a previously stored pattern. This activity triggers a pattern $\tilde{\text{q}}(0)$ in CA3 directly via the previously learned weights from EC to CA3. The pattern then runs through 15 activation cycles of the auto-associative network in CA3 while leaving the input from the EC clamped (we have verified that after 15 cycles the results have converged). In more detail, for the *t*-th cycle the activation of CA3 cell *i* is

$$\begin{array}{c} h_{i}\left(t\right)=\alpha \sum_{j=1}^{N_{EC}}w_{ij}\hat{p}+\beta \sum_{j=1}^{N_{CA3}}v_{ij}\tilde{q_{j}}\left(t-1\right), \end{array}$$

where *α* and *β* are constant factors set to 1 and 3 and $\tilde{\text{q}}(t)$ is determined by the k-WTA mechanism described in Eq 2. Hence, during recall CA3 activity is dominated by the recurrent connections and the DG is not involved anymore. The resulting pattern $\tilde{\text{q}}(15)$ triggers one in CA1, which in turn determines the output activity in the EC via the learned weights from CA3 to CA1 and CA1 to EC, respectively. In simulations without recurrent connections $\tilde{\text{q}}(0)$ activates a pattern in CA1 immediately.

### Evaluation

Memory performance is determined by the network’s ability to perform pattern completion. In more detail, after storage, patterns are presented to the network again, but now in a corrupted version called recall cue. If the network’s output is more similar to the original pattern than its cue was, then the network has done some amount of recall. As a measure for similarity we use the Pearson correlation coefficient. For instance, the correlation between the originally stored pattern **p** in the EC and the reconstructed one $\tilde{\text{p}}$ is defined as:

$$\begin{array}{c} Corr\left(p,\tilde{p}\right)=\frac{{\left(p-\bar{p}\right)}^{T}\left(\tilde{p}-\bar{\tilde{p}}\right)}{∥p-\bar{p}∥\cdot ∥\tilde{p}-\bar{\tilde{p}}∥}, \end{array}$$

where $\bar{p}$ and $\bar{\tilde{p}}$ are the means of **p** and $\tilde{\text{p}}$, respectively. The higher this correlation is, the more similar is the recalled pattern to the original one. Furthermore, we define the average correlation over all stored patterns {**p** ^(*s*)^:1 ≤ *s* ≤ *M*} as

$$\begin{array}{c} Corr_{EC}=\frac{1}{M}\sum_{s=1}^{M}Corr\left(p^{\left(s\right)},{\tilde{p}}^{\left(s\right)}\right). \end{array}$$

We perform simulations where we alter the quality of the recall cue and we illustrate the memory performance by plotting *Corr*_*EC*_ over the quality of the cues, i.e. the average correlation the cues have with the original patterns. Measurements above the main diagonal show then that the output of the network is on average more similar to the stored patterns than the cues. Hence, the more the measurements are above the diagonal, the better is the performance. To investigate how much pattern completion each subregion contributes to the overall performance, we similarly define *Corr*_*CA*3_ and *Corr*_*CA*1_.

### Different Networks

We compare the model to two alternatives. Firstly, to determine how effective the CA3 recurrent connections are, we perform simulations of a network without these connections. Here, the pattern $\tilde{\text{q}}(0)$ is directly transferred to CA1 during recall without undergoing the activation cycles of the auto-associative network in CA3. The result of these simulations are indicated by dashed lines throughout the figures.

Secondly, we investigate the ability of a minimal EC-CA1-EC model to store patterns. We performed simulations, in which during storage, activity in CA1 is triggered by the input from EC-CA3-CA1 pathway, without any plasticity in these connections. The CA1 patterns are then hetero-associated with the original input patterns in the connection weights EC-CA1 and CA1-EC, so in contrast to previous simulations the EC-CA1 connections are now plastic. During the recall phase, the recall cue is transferred to CA1 via the temporoammonic pathway and from there back to EC. The result of these simulations are indicated by magenta lines throughout the figures.

Besides outlined architecture, parameters do not change across simulations except in section ‘Comparison to the Model in Rolls (1995)’. All parameter changes there are described in the main text.

### Pattern Separation Index

To quantify the degree of pattern separation by the DG we plot the pairwise correlations of stored patterns in CA3 over the ones of the stored input patterns themselves and calculate the regression line between them. Whenever the line approximates the data well, then its slope is a good measure of how effective the DG separates the patterns. The flatter it is, the better is the separation. Thus, we refer to it as the pattern separation index.

## Results

### Comparison to the Model in Rolls (1995)

In a series of studies, a hippocampal model for memory formation within the standard framework has been established and tested computationally [5]. The main argument of this model is that CA3 equipped with many recurrent connections functions as an auto-associative network and is the crucial place for pattern completion. To test the theory, performance of simulations where those connections have been removed, has been compared to performance of the full network.

**Fig 2:**
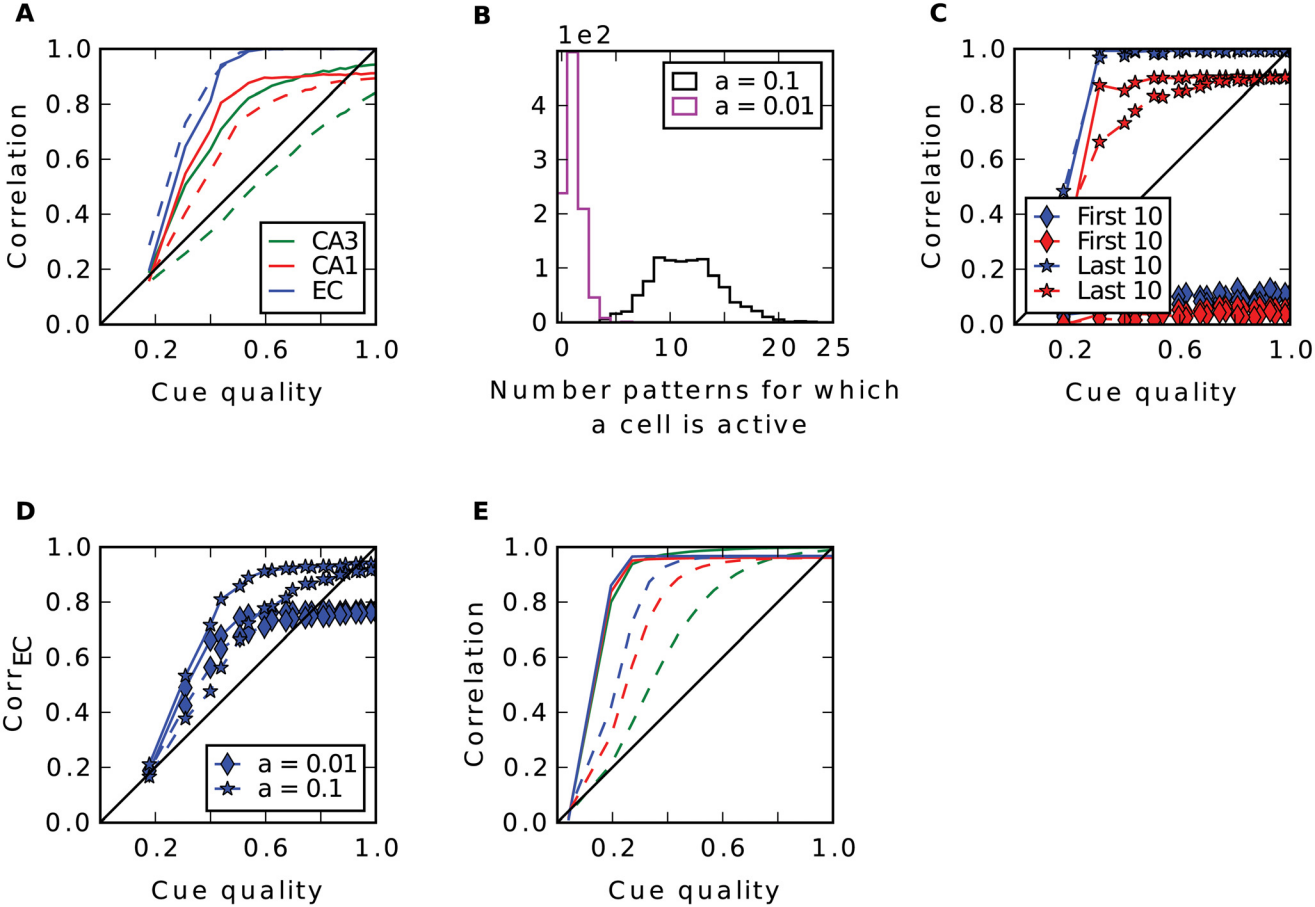
Analysis of the model by Rolls (1995). **A**: Recall performance in the model as proposed in [5]. Different colors show mean correlation between reconstructed patterns and stored ones in different regions; dashed lines show performance in a simulation where the recurrent connections in CA3 were turned off. **B**: Histogram of CA1 cell firing during storage. When sparsity is 0.01 (magenta) each cell fires about one time. This grandmother-like coding is abandoned if sparsity is 0.1 (black). **C**: Recall performance in CA1 (red) and EC (blue) for sparsity 0.1 measured for the last 10 patterns stored (stars) and for the first 10 (diamonds). Abandoning the grandmother-like code leads to a breakdown in performance by forgetting previously stored patterns. **D**: Recall performance in EC when connectivity from CA1 to EC is not complete and sparsity in CA1 is 0.01 (diamonds). A grandmother-like code cannot reproduce the whole pattern if the connectivity is sparse. When CA3-CA1 is a hetero-association with sparsity 0.1 (stars) diluting the connectivity has a milder effect. **E**: Our model as described in text yields similar results as in **A**, but is biologically more plausible, we believe.

**Fig 3:**
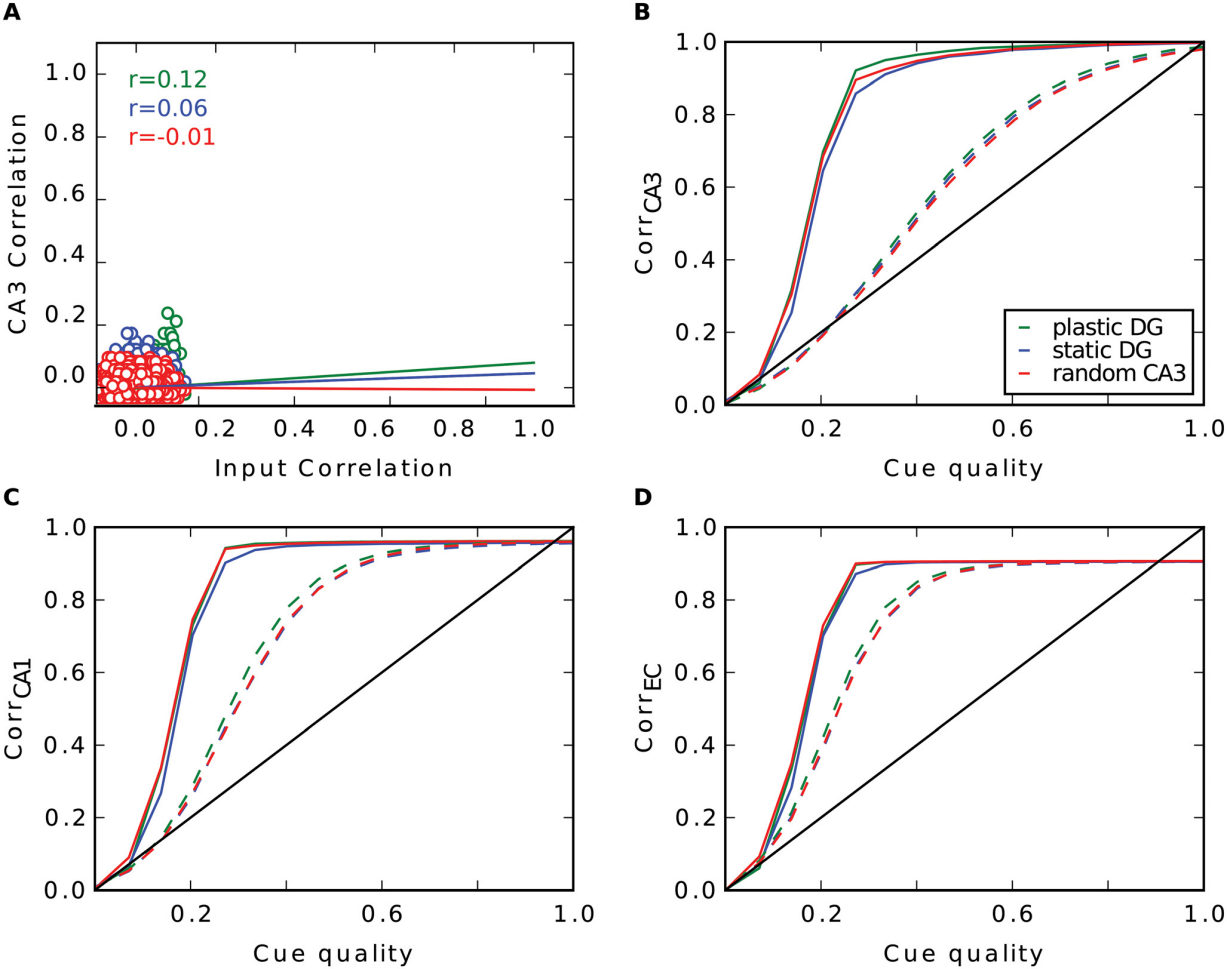
Pattern separation in the DG with random input. **A**: Pairwise correlation between stored patterns in CA3 as a function of pairwise correlation in EC with learning in DG (green), without (blue) and when the CA3 code is set up randomly (red). Regression lines are plotted, r values are shown in the upper left. **B-D**: Recall performance of the different simulations in CA3 (**B**), CA1 (**C**) and EC (**D**). Dashed lines are simulations where the recurrent connections in CA3 have been removed.

To reproduce the results of Rolls (1995) we performed a simulation of this model using the same parameters as in that study, including number of cells and connections and the sparseness parameter, and stored 100 random patterns. Fig 2A shows the average correlation between stored patterns and the reconstructed ones in the EC vs. the cue quality. Since the curve is well above the diagonal the network as a whole performs pattern completion. Only when the cue quality becomes highly degraded, pattern completion starts to break down. The intermediate stages of the network, CA1 and CA3, while not as efficient as the entire network, perform pattern completion as well to a certain degree (Fig 2A). To specifically test the role of the recurrent connections in CA3, we performed the same analysis without those recurrent connections. In this case, pattern completion in CA3 was abolished (Fig 2A, dashed green line). However, as in the data of Rolls (1995), at the output level pattern completion was not affected. This has not been discussed and we will turn to this in more detail below. In conclusion, we reproduce the main results of the model (compare Fig 2A with [5, Fig 3 bottom]).

While most of the parameters in the Rolls’ model are consistent with the rat hippocampal anatomy, two clearly are not. Firstly, in the model CA1 the sparsity, i.e., the proportion of cells being active, *a*_*CA*1_ = 1%, is much lower compared to the other regions, but the contrary is true in the real rat hippocampus [24, 34] (and see sparsity estimates in Methods). This way, many CA1 cells only code for one pattern as shown in Fig 2B and the pattern a cell codes for is burned into the weights of that cell, which is reflected in a high learning rate in CA1. However, it is unrealistic to assume such a coding scheme. Since it allows CA1 to store only $\frac{1}{a_{CA1}}=100$ patterns, even when numbers of cells and connections are scaled up to realistic ones of several hundreds of thousands as in the rat. This sparse coding scheme is functional, since the recall performance breaks down when we abandon it by increasing the sparsity to 10% (Fig 2C). In particular, patterns that are stored in the beginning of learning are overwritten by patterns that are stored later.

Secondly, full connectivity from CA1 to EC is assumed. This property is important, too. When the connectivity is diluted like between other regions, the low activity in CA1 is unable to trigger the whole original pattern in the EC (Fig 2D, diamonds). In this case, given a pattern in CA1, due to its high sparsity there are a few cells in EC that do not get any activation from it. However, this high connectivity is biologically not plausible.

To improve on these two inconsistencies we propose that, during storage, CA1 is activated by the EC via the temporoammonic pathway, that has not been considered yet. Thus, rather than a competitive one shot learning, we suggest a hetero-association between CA3 and CA1 as between EC-CA3 and CA1-EC. Now, the network recalls well even when the connectivity is not complete and the sparsity in CA1 is not unreasonably high (Fig 2D, stars). An alternative could be to keep the one shot learning and lower the sparsity and the learning rate. However, for simplicity we choose the former option.

Besides the changes in CA1, we scaled the model up and adjusted all parameters to more biological plausible ones (Fig 1A) and simplified the activation function to a k-WTA mechanism (see Methods for details). Overall, these changes did not alter the behaviour of the network (Fig 2E), although the presence of recurrence in CA3 now has a stronger effect on pattern completion at the output stage. Notice also, that the completion of the first hetero-association from EC to CA3 is much more effective. Due to a very sparse coding in Rolls’ EC (5%) and a sparse connectivity the signal cannot be transferred properly to CA3 during recall. This is not the case in our model, since here the sparsity in EC is 35%.

From now on, all simulations are performed with continuous input, thus the model is now as described in the Method Section.

### Pattern Separation in DG

The standard framework suggests that the role of the DG is to perform pattern separation [7–9]. This process transforms correlated patterns in the EC into more uncorrelated ones in CA3. This is a necessary operation, since a Hopfield-like auto-associative memory in CA3 would only be efficient in storing patterns that are nearly orthogonal to each other [35]. [5] has suggested that pattern separation can be learned by a Hebbian competitive net, however, that has not been verified computationally. We therefore investigated whether DG is a good pattern separator and whether Hebbian learning enhances this function. We compared three different simulations. One with learning in the DG enabled, one where it is disabled, and one simulation, where we modeled the DG as a perfect pattern separator. In the last case, we removed the EC-DG-CA3 pathway and instead artificially set up a random uncorrelated code in CA3 for storage. Each set of simulations were performed with random input and more realistic grid cell input (see Methods).

As one might expect, with random input there are no great differences in performance between the three simulations (Fig 3B–3D). Patterns in the EC input are already uncorrelated by construction. This low degree of correlation is then just transferred to CA3. Hebbian learning in connections between EC and DG is not able to remove any more correlation (Fig 3A). Since the pairwise correlation in CA3 is not linearly dependent on the ones in EC (r value ranges from -0.01 to 0.12), the pattern separation index is not reliable here.

**Fig 4:**
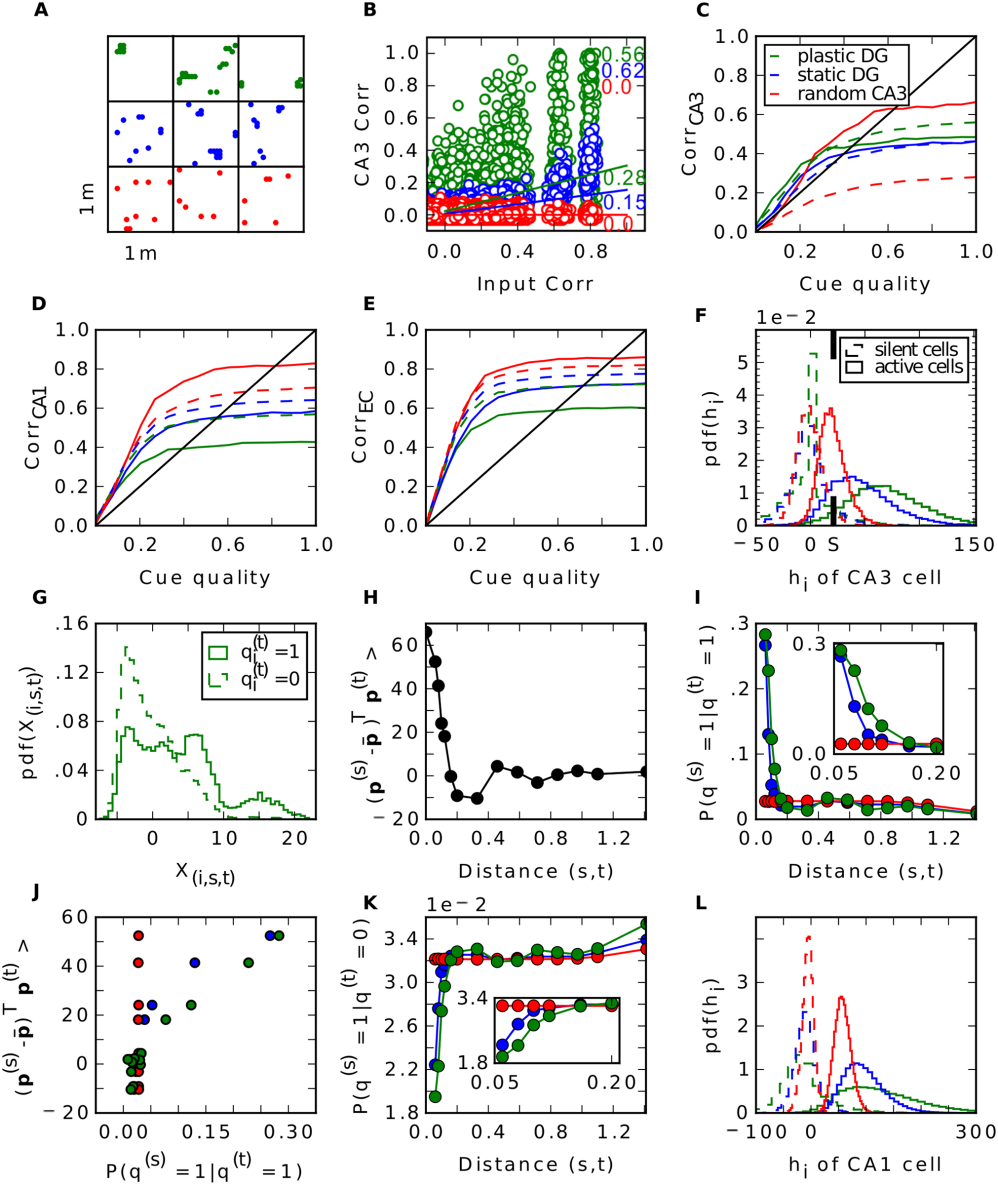
Pattern separation in the DG with grid cell input. **A**: Firing of three typical CA3 cells across all stored patterns plotted over the environment. Colour code as in Fig 3. **B**: Pairwise correlation between stored patterns in CA3 as a function of pairwise correlation in EC. Number next to regression line show its slope, r-values are shown in the upper right. **C-E**: Recall performance of the different simulations in CA3 (**C**), CA1 (**D**) and EC (**E**). Dashed lines are simulations without recurrent connections in CA3. **F**: Distribution of activities during recall when a cell fires during storage (solid) or is silent during storage (dashed) in CA3 when noiseless cues are given. *S* = ⟨*S*_*t*_⟩_*t*_ indicates the average signal term in Eq (5). **G**: Distribution of crosstalk terms of cells that fire (solid) and are silent during storage (dashed). **H**: Average overlap of two pattens **p** ^(*s*)^ and **p** ^(*t*)^ in the EC plotted over the distance of *s* and *t*. **I**: Probability that a CA3 cell fires at a location s given it fires at t plotted over the Euclidean distance of s and t. Inset shows zoomed plot. **J**: Average Overlap of two pattens **p** ^(*s*)^ and **p** ^(*t*)^ in the EC plotted over the probability a cell fires at *s* given it fires at *t*. **K**: Probability a cell fires at s, given it is silent at t. Inset shows zoomed plot. **L**: Same as **F** but in CA1.

By contrast, with grid cell input from the EC, Hebbian learning has a strong effect on the network. One observation is the different firing behaviour of CA3 cells. Since each input pattern refers to one location in space, we can illustrate the firing of CA3 cells over all stored patterns plotted over the environment (Fig 4A). Note that only 252 of the 400 locations can be occupied, as only 252 patterns were selected for storage. We observe that after learning, many cells in CA3 establish place fields. They fire around certain locations, but are silent elsewhere. This is in accordance to other work that have shown that Hebbian learning indeed transforms grid cell code into a place field representation [36–40]. Consequently, the probability a CA3 cell fires at location *s* given it fires at location *t* is significantly higher when the Euclidean distance between these locations is small than when they are far away (green line in Fig 4I). When learning is disabled, a typical cell in CA3 fires scattered over the entire space and is more comparable to a CA3 cell that is created randomly, as in the third simulation. Hence, the probability it fires at *s* is no longer dependent on the distance to *t* in the random CA3 case (red curve in Fig 4I). This dependency is weaker when the DG connections are static (blue curve). In particular, the dependency extends to a smaller radius.

More interestingly, we find that Hebbian learning does not support pattern separation. To the contrary, we have measured the pairwise correlation between all stored patterns in CA3 and in EC and we have found that some patterns are highly similar in CA3 (Fig 4B) when learning is enabled. This is a direct consequence of the established place-field-like code in CA3. Patterns referring to close locations are very similar. Without learning, we do not see patterns of such high correlation, since CA3 cells are not as spatially selective as before. This is in line with the lower pattern separation index of the static DG (0.15) compared to the plastic one (0.28). In the simulation where the DG is modeled as a perfect separator the correlation between two patterns is distributed around zero and no high correlations are found by definition.

In simulations without recurrent connections, the consequence of a place-field-like code in CA3 is a better recall performance in CA3 compared to the other scenarios, but a worse one at the output level in the EC (Fig 4C–4E, dashed lines). To investigate the reason for the improvement in CA3 in this simulation, we looked at the activity distributions of cells during recall with a noiseless cue. We distinguished between activities of cells that should fire given the present recall cue and those of cells that should be silent. To no surprise, we find that the mean of the former is much higher. With plasticity in the DG the two distributions have very little overlap (green curves in Fig 4F). Thus, it is very rare that a cell that should be silent receives more activation than a cell that should be active. Hence, very few mistakes are made. In contrast, if the CA3 code is random, these distributions overlap more and false behaviour occurs more often.

What is the origin of this effect? In Eq (5) we expressed the activation of cell *i* given the noiseless recall cue **p** ^(*t*)^ as the sum of the signal *S*_*t*_ and the crosstalk terms. In the random case, the activations of cells that should be silent are distributed around 0. Here, in each activation the signal term vanishes (because $q_{i}^{\left(t\right)}=0$) and it is only influenced by the sum of crosstalk terms. For the activations of cells that should fire, the signal term does not vanish (because $q_{i}^{\left(t\right)}=1$) and the distribution is shifted to the right by the average signal *S* = ⟨*S*_*t*_⟩_*t*_ while its shape is preserved (we find that the variance of *S* is negligible). Hence, the sums of crosstalk terms are not dependent on whether a cell fired during storage or not.

In contrast, in a place-field-like code these distributions are not just shifted by *S*. Here, crosstalk terms tend to be larger, when a cell is supposed to fire (Fig 4G). Note that each crosstalk term *X*_(*i*,*s*,*t*)_ is proportional to $q_{i}^{\left(s\right)}$, the firing of cell *i* at location *s*, times the overlap ${\left({\text{p}}^{\left(s\right)}-\bar{\text{p}}\right)}^{T}{\text{p}}^{\left(t\right)}$ of the input pattern at location *s* with the cue. Suppose cell *i* has fired at *t*, as seen in Fig 4I, $q_{i}^{\left(s\right)}$ is more likely to be non zero when location *s* is nearby location *t*. Additionally, due the spatial character of the grid cell input, the overlap is highly dependent on the distance, too, and is maximal when the locations are close by (Fig 4H). Thus, the more likely $q_{i}^{\left(s\right)}=1$, the higher is the overlap as shown in Fig 4J (green dots). This is not true when cell *i* has been silent at *t* (Fig 4K). Here, the cell is less likely to fire at nearby locations and hence crosstalk terms with a large overlap factor do vanish at least as often as others. Therefore, crosstalks are greater in cells that should be active than in cells that should not. This explains the higher activation of cells that should be active and the better performance.

When learning is disabled the probability of $q_{i}^{\left(s\right)}$ is less dependent on the distance of *s* and *t*. Hence, the relation of the overlap with $q_{i}^{\left(s\right)}$ is less pronounced (blue dots in Fig 4J). Consequently, this relation disappears in a random CA3 code, since here a CA3 cell fires entirely independently on the distance (red dots in Fig 4J).

The advantage in performance when learning is enabled, however, is already gone at the CA1 stage (dashed lines in Fig 4D). Due to the high similarity of some patterns in CA3, some crosstalk terms in CA1 become very large. The consequence is a high variance of the sum of crosstalks and hence wider distributions of activities of cells that should be active and of those that should not. This results in a high overlap between these two distributions, thus many errors are made (Fig 4L).

Without Hebbian learning in the DG and in the simulation where the DG is a perfect separator, we do not see this high variance because of the lack of patterns that are highly similar. Here, the distributions are sharper resulting in less overlap and fewer mistakes (Fig 4L).

Furthermore, the recall correlations in CA3 without recurrent connections are very low, in particular when the CA3 patterns are created randomly (red dashed line in Fig 4C). This requires an explanation.

Even though the 252 patterns stored in CA3 are orthogonal and span a high dimensional space, due to the high correlations in the grid input, the learned EC-CA3 weights, span a much lower dimensional space. When CA3 patterns are projected into this low-dimensional subspace, the correlation between recalled and stored patterns are high, i.e., the EC-CA3 hetero-association works in principle. However, when assessing the retrieval quality, we compare the retrieved to the stored pattern in the larger dimensional space of CA3 patterns. Since the EC-CA3 weights span a low-dimensional space, they cannot address the higher dimensional space and, therefore, the correlations between stored and recalled patterns are low, and the dashed red line in Fig 4C is far below the diagonal.

The recurrent collaterals in CA3 are doing their job well in the random CA3 case since the solid red line is well above the dashed one in Fig 4C. However, the solid red line is still barely above the diagonal for low to moderate cue quality and well below for high cue quality, because the auto-associative net cannot entirely overcome the limitation of the EC-CA3 projections. As in the network without recurrent connections, when patterns are projected onto the low-dimensional subspace, recall performance would be much better. That information about the stored input patterns is preserved in CA3, despite the low retrieval correlations, is evident when examining the later stages of hippocampal processing, in CA1 (Fig 4D) and EC output (Fig 4E). There, the retrieval performance is quite high for random CA3 patterns. The fact that it is better than for the static or plastic DG case confirms that auto-associative networks perform best for uncorrelated (CA3) patterns.

For the static and plastic DG case, we find that without recurrent connections performance is better than for random CA3 patterns (Fig 4C, green and blue dashed lines lie above red dashed line) and that the difference between recurrent and no recurrent connections is less pronounced (compare respective solid to dashed lines). These findings lend further support to our explanation for the retrieval correlations for random CA3 patterns. When DG is static or plastic, the pattern in CA3 is driven to a certain extend by the EC input (via DG) and thus is correlated with it. Therefore, the mismatch between the dimensionality of the CA3 patterns and that of the EC inputs is lower and, as a result, the retrieval correlations in CA3 are higher. However, the correlations between stored CA3 patterns reduce the ability of the CA3 recurrent network to perform pattern completion, which hurts retrieval performance in the downstream layers (Fig 4D and 4E).

To summarize, Hebbian plasticity does not enhance pattern separation as suggested in [5]. When grid input is given, it has even the contrary effect and hence harms memory performance. Moreover, we find that a static DG performs decent pattern separation. Therefore, in the following analysis learning in the DG is disabled.

### Pattern Completion in CA3

To test the hypothesis that CA3 functions as an auto-associative memory, we compared a simulation of the complete network with one, where we disabled the recurrent connections. Once again, we perform this comparison for random and grid cell inputs.

**Fig 5:**
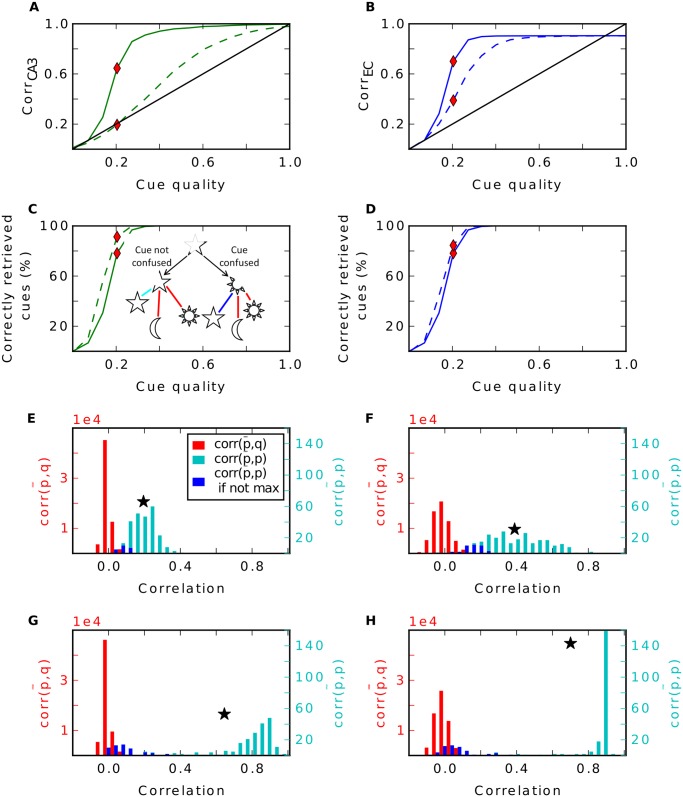
Recall performance of the model with random input. Performance in CA3 (left column) and in EC (right column). **A-B**: Recall performance in CA3 and EC; dashed lines are simulations without recurrent connections. **C-D**: Proportion of correctly retrieved cues. Left side in inset of **C** illustrates that the cue (star) is retrieved correctly. The reconstruction is most correlated with corresponding stored pattern (star) compared to the other stored pattern (moon and sun). Right side shows when cue is confused with another stored pattern. Here the reconstruction is more correlated with the sun than with the star. Coloured bars explain code in **E-H**. **E-F**: Histogram of pairwise correlations between reconstructed pattern and corresponding stored pattern (cyan, blue) and between reconstructed pattern and another stored pattern (red). Blue indicate the cases when the correlation between the reconstructed pattern and the stored pattern is not maximal. Star marks mean of the distribution of the correlation between the reconstructed pattern and the stored pattern. The histogram is calculated at the cue quality indicated by the red rhombus in **A-D**. **G-H**: Same as **E-F** but with recurrent connections enabled.

When random inputs are presented, we indeed find the recurrent connections performing a fair amount of pattern completion in CA3 (Fig 5A) as also found by [5]. At the output stage in the EC, however, the recurrent connections in CA3 are only beneficial when cues are highly degraded. Both simulations with and without recurrent CA3 connections perform equally well for strong to moderate cue qualities (Fig 5B). Thus, in these cases the hetero-associative steps EC-CA3, CA3-CA1 and CA1-EC are already sufficient for pattern completion.

One can argue that a good recall performance does not only include a high correlation between reconstructed and original pattern, but also require that the recalled pattern is more similar to the original one than to any other one. To investigate this, we compared the correlation between a reconstructed pattern and its original stored version with the correlations between this reconstructed pattern and all other stored patterns. If the pattern is remembered correctly, the former correlation should be larger than all of the latter ones. Otherwise, the recall cue has been reconstructed closer to a wrong pattern and hence it has been confused by the network as another stored pattern (see inset of Fig 5C for illustration). We find that the simulations using the recurrent connections do confuse patterns more often when cue qualities are poor than do simulations without recurrent connections (Fig 5C–5D). At moderate to high cue qualities, the performance is equal with and without recurrent network.

In more detail, the mean of the distribution of correlations between the reconstructed patterns and their original version is increased by the recurrent connections, which is good. However, at the same time, this distribution becomes wider and even bimodal. Thus, it begins to intersect with the distribution of correlations between the reconstructed patterns and all other stored pattern. Consequently, it starts to confuse reconstructed patterns with the other stored ones (Fig 5G). This confusion cannot be solved at later stages and the correlation between these patterns and their originals stays low till the output stage (Fig 5H). The result is a bimodal distribution of correctly remembered patterns with high correlation and false recalled ones with correlation around 0. When the recurrent connections are disabled, the distribution of correlations in the EC stays unimodal with a lower mean but fewer patterns are confused (Fig 5F).

To summarize, for moderate to good cue qualities, the computation of the recurrent connections is completely redundant, since the pattern completion is also performed by the inevitable decoding pathway over CA1. For weak cues, the recurrent connections do help recall, but this advantage comes at the price of a higher confusion rate.

**Fig 6:**
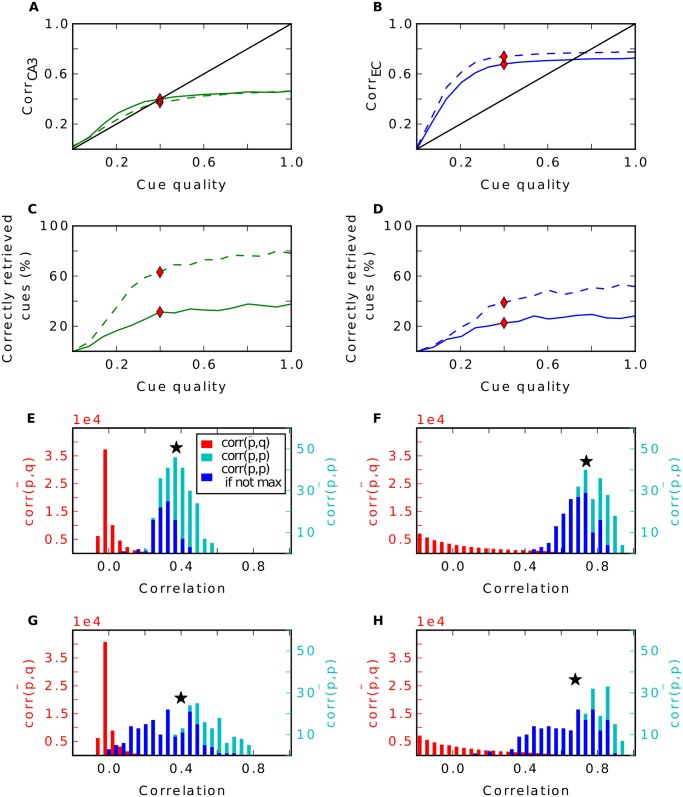
Recall performance of the model with grid input. Effect of recurrent connections when grid cell input is given. Plotting conventions as in Fig 5.

We also tested how effective the pattern completion by the recurrent connections is in the grid cell input scenario. We observe that having these connections helps in CA3 only marginally, but at the price of a significantly higher confusion rate (Fig 6A–6H). More importantly, at the output level in EC the recurrent connections become a deficiency for the model and the performance is worse. Additionally, the higher confusion rate is still apparent. Thus, the recurrent connections are not only redundant but even harmful for memory performance for all cue qualities.

### Recall by the EC-CA1-EC Pathway

**Fig 7:**
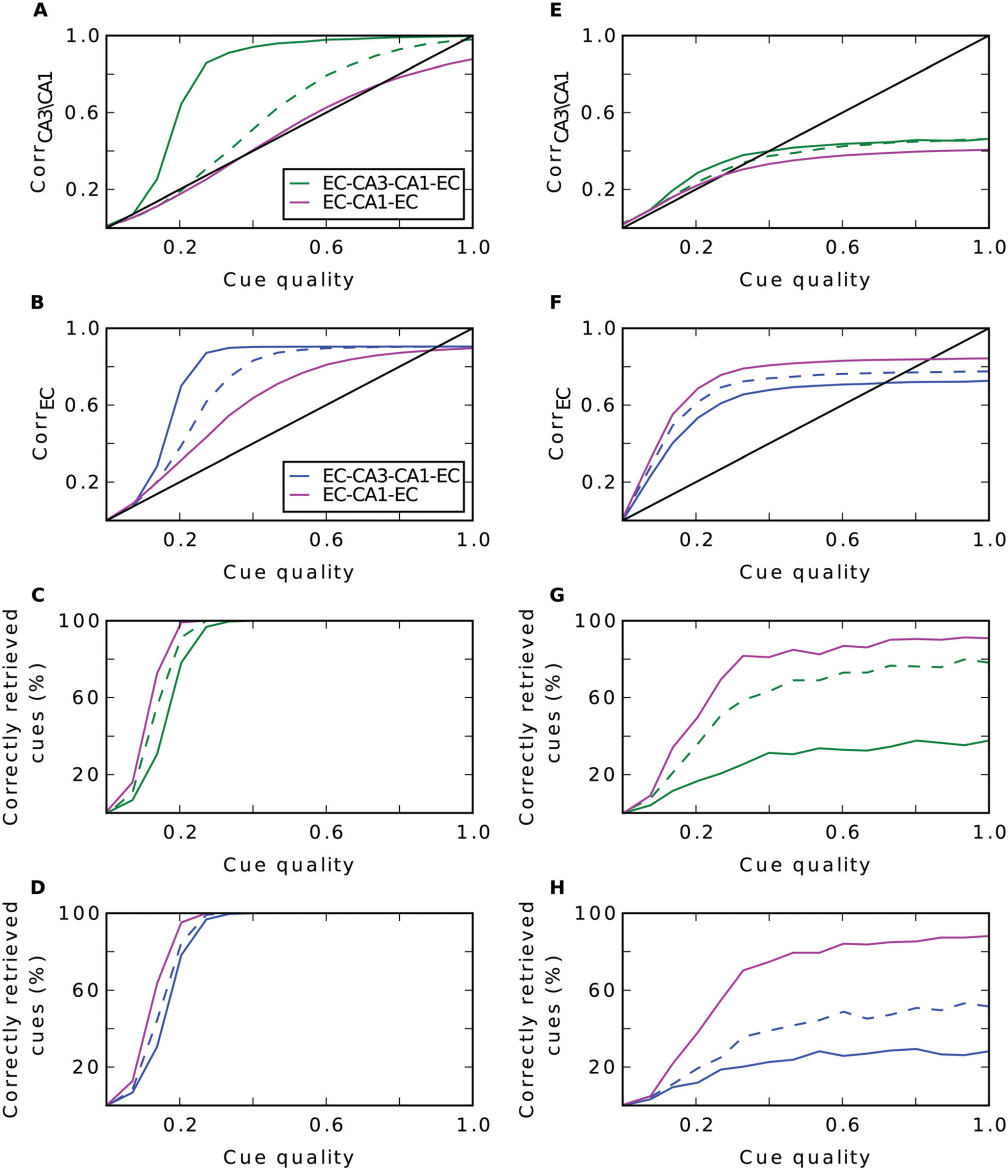
Comparison of the complete model with the simpler EC-CA1-EC model. Performance of the complete model (green and blue) and of the simpler EC-CA1-EC model (magenta) when random input is given (**A-D**) and grid input is given (**E-H**). **A**: Recall performance in CA3 (complete model) and CA1 (simpler model). **B**: Performance in EC. **C-D**: Proportion of correctly retrieved patterns in CA3/CA1 (**C**) and EC (**D**), respectively; dashed lines are simulations without recurrent connections. **E-H**: Same as left column, but with grid cell input.

An alternative proposal is that pattern completion is performed by the pathway EC-CA1-EC [13]. Our data shows that recurrence in CA3 is redundant and that three hetero-associations are sufficient for completion. We investigated, whether the two associations EC-CA1-EC are sufficient for pattern completion as well. The results are shown in Fig 7.

When input patterns are created randomly, the simpler model confuses fewer patterns (Fig 7C–7D), but performance in terms of correlation is worse than with the complete network (Fig 7A–7B). It seems that in this scenario two steps are not sufficient to reconstruct the whole pattern. Interestingly, in the more realistic grid cell input scenario, the two steps in the alternative model are slightly more effective in pattern completion than the complete network (Fig 7E–7F). Moreover, the former confuses far fewer patterns than the latter (Fig 7G–7H).

We wondered whether scaling factors effect the model. Thus, we performed simulations as in Fig 7 where we scaled down the number of neurons by only 20 rather than by 100. We stored 252 ⋅ 5 patterns while leaving all other parameters constant. In particular, the number of synapses is still scaled by 10. We find no qualitative differences between the simulations indicating that our results do not change when numbers of cells and synapses approach the realistic ones.

### LEC Input and Different Environments

**Fig 8:**
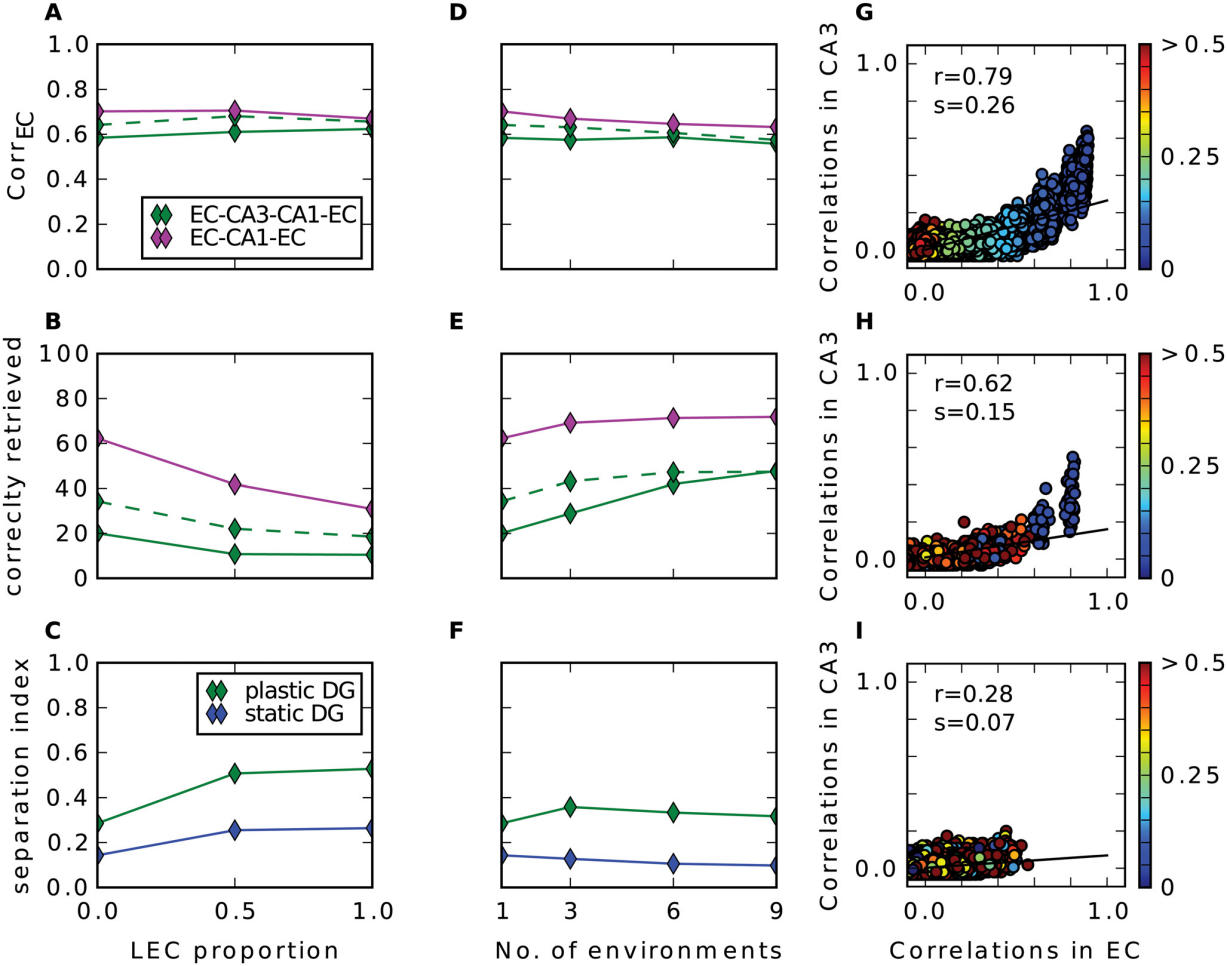
LEC input and multiple environments. Results of simulations with additional LEC input (**A-C**) and with input from multiple environments (**D-F**). First row (**A, D**) shows the recall correlation in EC averaged over all cue strengths, second row (**B, E**) shows averaged proportion of correctly retrieved patterns and last row (**C, F**) shows pattern separation index. **G**: Pairwise correlation between stored patterns in CA3 as a function of pairwise correlation in EC in a simulation with only LEC inputs. Euclidean distance (in m) of the pair is indicated by the colour of disk according to the colourbar. Black line is the regression line with slope s (separation index) and r value shown in the upper left. **H-I**: Same as (**G**) in a simulation with only MEC input originating from nine different environments. **H** shows all pairs where both patterns come from the same map, **I** show all pairs where the patterns come from different maps.

Up to now we have considered only input to the hippocampus that originates from the medial part of the entorhinal cortex. Studies have shown that substantial part of hippocampal input comes from the lateral entorhinal cortex (LEC). In contrary to the grid cells, neurons in LEC fire only weakly spatially modulated [41] and rather respond to individual objects [33]. How do the networks behave under the influence of such input? Since the proportion of LEC input relative to MEC input is not clear, we parametrized it and performed simulations with different proportions of LEC cells. We find that the recall correlations in EC are not affected much by adding LEC input (Fig 8A). When input comes only from LEC the networks confuse patterns more often. Because of the pseudo place field code in LEC, nearby patterns are highly correlated and the radius to which this extends is slightly larger than in the grid cell code ((compare Fig 8G with Fig 4B)). Consequently, the number of high correlated patterns is higher which results in a higher confusion as well as in a slightly less effective pattern separation.

Fyhn et al. (2007) found that when a rat is exposed to a new environment the grid cells remap, i.e. their grid pattern rotates and shifts coherently while the spatial frequency remains roughly stable [26]. We investigated how well the networks can store patterns of activity originating from different environments rather than from just one. We find that by increasing the number of maps, the recall correlations and the proportion of correctly retrieved patterns of the networks with and without recurrent connections become almost equal, where the EC-CA1-EC network remains the best in both measures (Fig 8D–8E).

As argued above, already a small number of moderately correlated patterns in CA3 degrades the auto-association and the subsequent hetero-association with CA1. Given just one map, correlation appears only in patterns that are nearby. We wondered, whether this is still true comparing patterns from different environments. In Fig 8H–8I we see the pairwise correlations of stored patterns in CA3 over the ones in the input in a simulation where we store patterns from nine different environments. Comparing patterns that originate from the same map, only those that are up to 5 cm apart have a high correlation above 0.6 and these are the only pairs, that remain to have moderate correlations left in CA3(Fig 8H). Many pairs, that are not nearby, have a fair correlation in the input but are almost uncorrelated after pattern separation through the DG. This can be observed for pairs where each pattern is from a different environment, too(Fig 8I). Many of them are moderately correlated in the input, but no longer in CA3. The remapping of the grids does not orthogonalizes the activities in the EC. Nevertheless, after pattern separation by the DG the patterns become almost uncorrelated in CA3.

To conclude, when patterns are stored from several environments, the relative number of pattern pairs that are nearby and from the same environment decreases and with it the number of pairs with remaining correlation in CA3. This benefits in particular the recurrent network and it performs as well as the network without recurrent connections. Nevertheless, the EC-CA1-EC network performance is best in all scenarios.

## Discussion

In the last decades, a view has evolved about how the peculiar anatomic structure of the hippocampus serves memory formation. It has been postulated that the CA3 region with its many recurrent connections functions as an attractor network performing pattern completion when degraded input is given [7, 11]. Thus, it is believed that CA3 is the actual storing place. Complex mathematical analysis show that the memory capacity of such a network is sufficient, when the activity in the region is sparse enough [30, 35, 42]. However, a decorrelation among the stored patterns is crucial and all the analysis supposes that. It is believed that the DG removes all correlations from the input patterns of the EC and performs the so called pattern separation. This is supported by a sparse coding, by strong and sparse synapses from DG to CA3 [9, 11], and by Hebbian plasticity from EC to DG during storage [5].

The present study challenges this view and shows several weaknesses of it. Firstly, in the mathematical analysis of the standard framework only an isolated CA3 network has been considered and the inevitable decoding pathway via CA1 has been neglected. We show that this pathway is capable of reconstructing the memory even when the recurrent connections in CA3 are removed. This makes the assigned auto-associative function of CA3 redundant for low to moderate noise levels. Interestingly, by arguing for CA3 being an attractor network, Treves and Rolls (1991) compared the ability of pattern completion of an auto-associative network with that of subsequent hetero-associative networks [42]. They conclude that when the sparsity of the activity approaches zero, the performance of a single auto-associative memory reaches nearly that of several hetero-associations, while at the same time fewer neuronal components are needed. However, in the standard model these components have to be present to perform encoding and decoding, turning this argument against the proposed function of CA3.

Secondly, simple Hebbian plasticity in the DG as proposed by Rolls (1995) does not support pattern separation. To the contrary. We have shown that due to this plasticity the grid cell code in the EC is mapped into a place field like code in CA3. This is in line with other work, that investigate the transformation from grid cells to place cells [36–40] by Hebbian learning. In this code, patterns from nearby locations happen to be highly correlated, which is the opposite of what a pattern separator should do. Thus, a competitive net is not a good candidate to orthogonalize patterns for grid cell input. What is not modeled here, is adult neurogenesis in the DG [43]. This additional plasticity might support pattern separation in contrast to Hebbian learning alone. Weisz and Argibay (2009) studied the effects of neurogenesis in the standard model and they find advantages in memory performance, but they only considered the case of random inputs [44]. However, alternative hypotheses for adult neurogenesis exist (e.g [18]). We show that by having random and fixed connections the DG performs quite well as a pattern separator. Only for very highly correlated patterns in the input, there remains some amount of correlation in these patterns after applying the separator. Despite the significant reduction, this amount is still enough to degrade the auto-associative CA3. Thus, to make the standard model work, a separator is needed that functions perfectly. However, assuming it exists, the benefit of a recurrent CA3 net would still be small compared to the EC-CA1-EC model if applied to grid cell input.

Thirdly, a further challenging argument against an auto-associative function of CA3 is the fact that the actual representations in the mammalian CA3 are far from uncorrelated. The vast majority of active pyramidal cells are place cells [45], thus activity patterns are correlated by nature. Storing such patterns of continuous place cell activity that occur in one environment in an auto-associative network leads to a continuous attractor or so called chart [46]. Every point in this chart refers to the neural representation of one location in the environment. A degraded input is then attracted towards a point on the chart and the network is indeed able to diminish the noise orthogonal to the attractor. However, it has been observed that many points are not stable [47] and drift along the attractor until they reach a fix point [48]. This means that many finally retrieved patterns are representations of the wrong location. Since we store correlated patterns in the auto-associative CA3 net, a continuous attractor is also formed in the present model. It can store a large number of patterns drawn from the grid map moderately well, however, drifting occurs in the recall. This drifting is already reduced, since the CA3 representations are binary rather than continuous [49], but still apparent as reflected by the high confusion rate of patterns when using the recurrent connections in CA3 (see Fig 6C). Papp et al.(2007) state that the drift may be much slower than pattern completion and hence storage of locations is still possible [49]. In other words, pattern completion is already done in the first update cycles in the attractor network. This is in agreement with our results. By viewing each hetero-associative step as one update cycle, pattern completion is performed by the three associative networks without loosing accuracy caused by the drift and the auto-associative function in CA3 becomes redundant.

Finally, given the structured grid cell input in the EC, the simpler network EC-CA1-EC is already sufficient for storage and pattern completion. Additionally, it confuses memories less often than the complete network does. This frees the recurrent CA3 connections to perform other functions. For instance, Levy (1996) suggested that CA3 associates its present activity with activity occurring in the past [50]. In this way, sequences of activities are stored which can explain the hippocampal involvement in tasks like trace conditioning or configural learning problems. A further alternative to an attractor net in CA3 has been suggested by Cheng (2013). Here, it is assumed that the recurrent CA3 network is not plastic, but rather creates intrinsic sequences which are then associated with temporal sequences of patterns in the EC [13]. We do not model temporal aspects here, but our study shows that because of the redundancy of CA3 as an auto-associative net, it very likely fulfils some other function. Similarly, the minimal model does not require plasticity in the synapses projecting from EC to CA3 nor in the Schaffer Collaterals, where plasticity has been found [51]. Hence, the plasticity could serve another function leaving the pattern completion in the model unaffected.

Experimental studies reported evidence for CA3 being an auto-association memory. For example, Gold and Kesner (2005) show that rats with lesioned CA3 are impaired in remembering a location when parts of the spatial cues are removed [52]. Another study obtains similar results when plasticity in CA3 synapses is corrupted in knock-out mice [53]. The authors conclude that CA3 is crucial for spatial pattern completion. In our opinion this is not convincing. If the actual storing place of the memory is CA3 then lesioning it or removing plasticity should give equivalent results as lesioning the entire hippocampus [13]. This is not the case, since in both studies animals behave normally in full cue conditions, but animals with hippocampal lesions are clearly impaired [54, 55]. An alternative interpretation for the experimental observations could be that the animals rely more on the integration of self motion cues in conditions when external cues are poor. This is in line with others who assign a path integration function to CA3 [46, 56]. If spatial information provided by the external cues is sufficient, spatial recognition can be performed by CA1 alone [57, 58].

Our work illustrates how essential it is to consider the whole hippocampal loop while investigating individual functional roles of the subregions.
